# Supplementary material for: Extensive diversity in RNA termination and regulation revealed by transcriptome mapping for the Lyme pathogen Borrelia burgdorferi
Source: Nat Commun. 2023 Jul 4;14:3931. doi: 10.1038/s41467-023-39576-1 (PMC10319736; doi:10.1038/s41467-023-39576-1)
Supplement: Supplementary file 1 — Supplementary Information [file 41467_2023_39576_MOESM1_ESM.pdf]

SUPPLEMENTARY INFORMATION

Extensive diversity in RNA termination and regulation revealed by transcriptome mapping for the Lyme pathogen *Borrelia burgdorferi*

Emily Petroni[1, †], Caroline Esnault[2, †], Daniel Tetreault[1], Ryan K. Dale[2], Gisela Storz[1] and Philip P. Adams[1,3,4,*]

[1]Division of Molecular and Cellular Biology, Eunice Kennedy Shriver National Institute of Child Health and Human Development, Bethesda, MD 20892, USA.

[2]Bioinformatics and Scientific Programming Core, Eunice Kennedy Shriver National Institute of Child Health and Human Development, Bethesda, MD 20892, USA.

[3]Postdoctoral Research Associate Program, National Institute of General Medical Sciences, National Institutes of Health, Bethesda, MD 20892, USA.

[4]Independent Research Scholar Program, Intramural Research Program, National Institutes of Health, Bethesda, MD 20892, USA.

[†]equal contribution

*correspondence: philip.adams@nih.gov

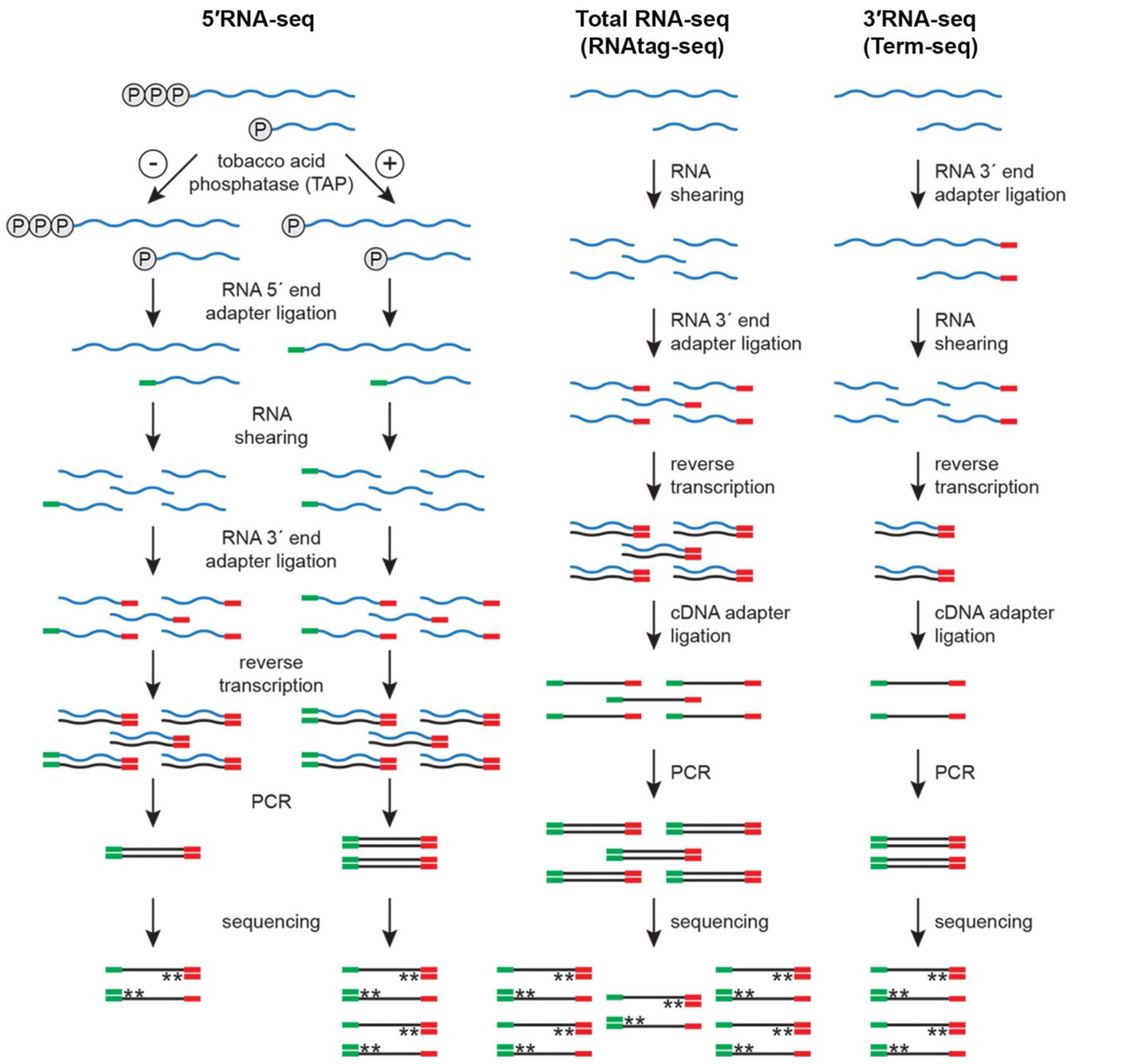

Fig. S1. Details of RNA-seq approaches. Schematic of 5′RNA-seq[1], total RNA-seq (modified from the RNAtag-seq methodology[2]) and 3′RNA-seq (modified from the Term-seq methodology[3]). 5′ phosphates (circled ‘P’), RNA 5′ end adapter or cDNA adapter (green line), RNA 3′ end adapter (red line), and stranded sequencing (asterisks) are indicated. Figure modified from[4].

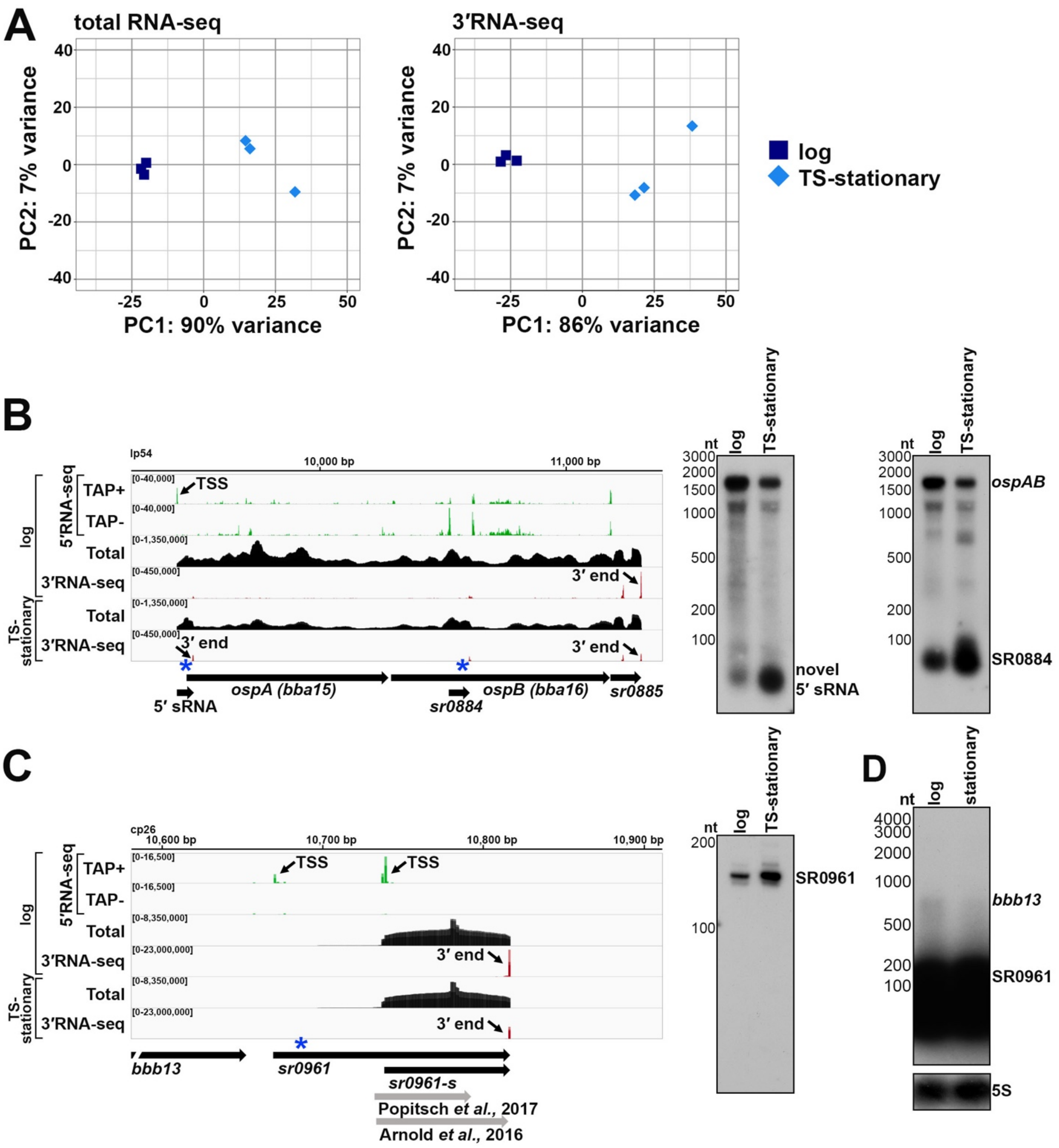

Fig. S2. Analysis of 3′RNA-seq replicates and additional northern analyses of *ospAB* and SR0961. **(A)** Principal component analysis plot to show correlation among total RNA-seq and 3′RNA-seq replicates. For each sample, total RNA-seq read counts or 3’RNA-seq 3′ ends were quantified at annotated genes and normalized by variance-stabilizing transformation.

Normalized counts were used in the principal component analysis to estimate the relationships between samples. RNA-seq browser image (left) and northern analysis (right) for the **(B)** *ospAB* locus and **(C-D)** SR0961 sRNA. Browser images display sequencing reads from logarithmic phase and TS-stationary phase cells, as in Fig. 2. The northern analysis in panel B was performed on the same blot from Fig. 2A; the northern analysis in panel C was performed on the same blot from Fig. 2E; the northern analysis in panel D was performed on the same blot from Fig. 6 and cropped to only show the logarithmic and stationary phase samples (RNAs were probed sequentially on the same membrane). The approximate probe sequence location is indicated by the blue asterisk on the corresponding browser image; **(D)** uses the same probe as in Fig. 2E, the membrane was also probed for 5S as a loading control. Size markers are indicated. A previously unannotated fragment is denoted as 'novel 5′ sRNA'.

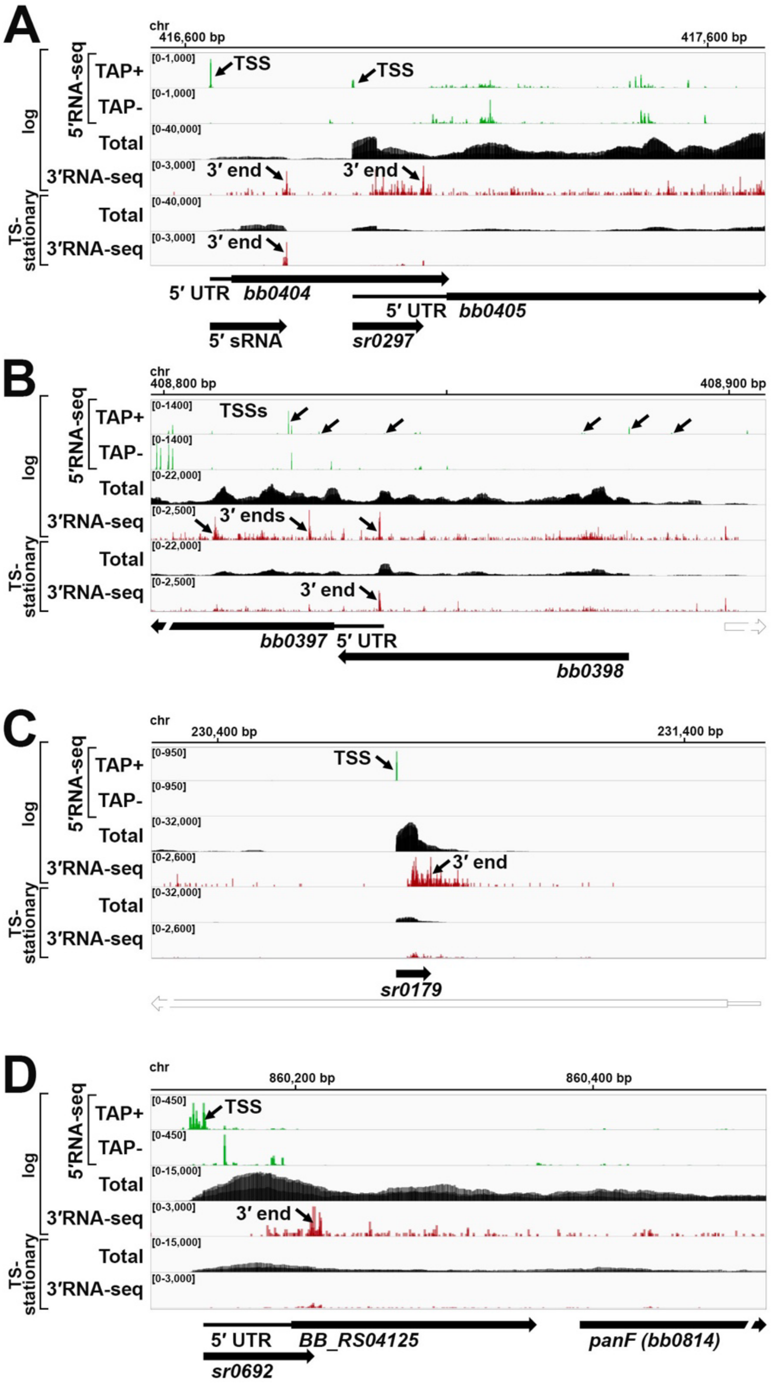

Fig. S3. Examples of complex or ambiguous annotations. RNA-seq browser image for the **(A)** *bb0404-bb0405* locus, **(B)** *bb0398-bb0397* locus, **(C)** SR0179 locus, and **(D)** *BB_RS04125* and *panF* locus are shown. Browser images display sequencing reads from logarithmic phase and TS-stationary phase cells, as in Fig. 2.

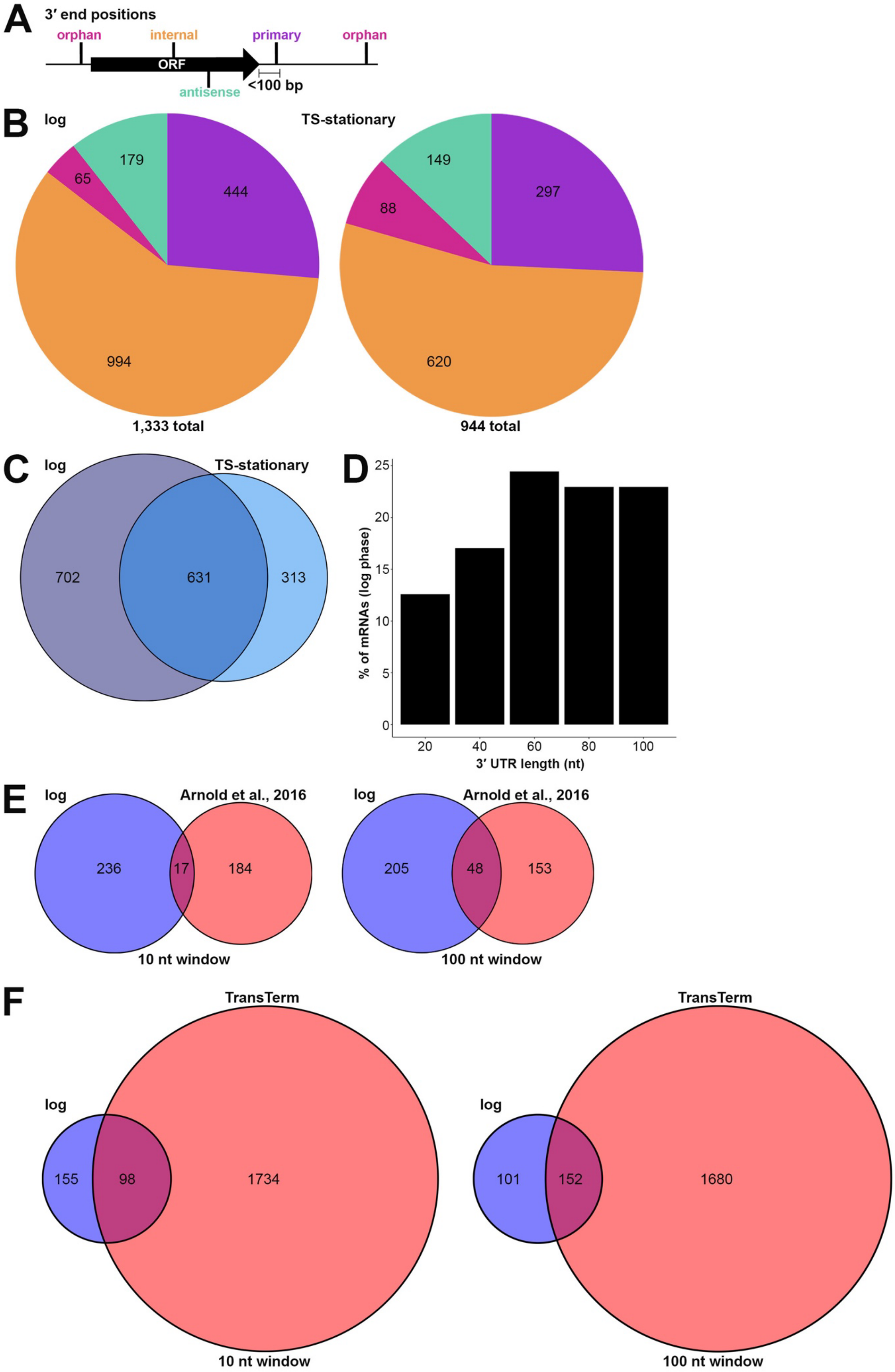
A
3′ end positions
orphan
internal
primary
orphan
ORF
antisense
<100 bp
B
log
TS-stationary
179
65
444
994
1,333 total
149
88
297
620
944 total
C
log
TS-stationary
702
631
313
D
% of mRNAs (log phase)
3′ UTR length (nt)
0
5
10
15
20
25
20
40
60
80
100
E
log
Arnold et al., 2016
236
17
184
10 nt window
log
Arnold et al., 2016
205
48
153
100 nt window
F
TransTerm
log
155
98
1734
10 nt window
TransTerm
log
101
152
1680
100 nt window

Fig. S4. Distribution of 3′ ends and comparisons to other studies. **(A)** Schematic of classification of 3′RNA-seq identified ends relative to an annotated ORF. 3′ ends were defined as: primary (purple colored, located on the same strand within 100 bp downstream of the 3′ end of an annotated gene (mRNA ORF, tRNA, rRNA, or sRNA)), antisense (aquamarine colored, located on the opposite strand within 50 bp of a gene start and end coordinates), internal (orange colored, located on the same strand within a gene) and orphan (fuchsia colored, located in a 5′ UTR, long 3′ UTR or not falling in any of the previous classes). The black arrow represents an ORF. **(B)** Distribution of 3′RNA-seq detected ends relative to annotated genes for logarithmic and TS-stationary phase conditions. Some 3′ ends fit the criteria for two different categories because of gene proximities; in the logarithmic phase condition: 100 are primary and antisense, 216 are primary and internal, 15 are internal and antisense, and 9 are primary, internal and antisense; in the TS-stationary phase condition: 64 are primary and antisense, 113 are primary and internal, 19 are internal and antisense, and 7 are primary, internal and antisense. **(C)** Comparison of 3′ ends identified in logarithmic phase culture to those identified in TS-stationary phase culture (Supplementary Data 1). If 3′ ends were called within a 10 nt window on the same DNA strand between both datasets, they were considered shared across those datasets. **(D)** Distribution of predicted logarithmic phase 3′ UTR lengths for annotated mRNAs. The distance between the annotated stop codon and primary 3′ end for a total of 152 mRNAs are presented by intervals of 20 nt. **(E)** Comparison of current 3′RNA-seq to total RNA-seq data from Arnold et al. 3′RNA-seq, logarithmic phase condition. Detected 3′ ends with an intrinsic termination score ≥ 3.0 (Supplementary Data 1) were compared to previously predicted *B. burgdorferi* intrinsic terminators[5] (Arnold et al. – Table S8, 'Predicted intrinsic terminators'). **(F)** Comparison of current 3′RNA-seq to terminators identified *in silico* by TransTerm. 3′RNA-seq, logarithmic phase condition. Detected 3′ ends with an intrinsic termination score ≥ 3.0 (Supplementary Data 1) were compared to predicted *B. burgdorferi* intrinsic terminators using the TransTerm terminator prediction algorithm[6]. For panels E and F, if predicted terminators were called within

a 10 or 100 nt window on the same DNA strand between both datasets, they were considered shared across those datasets**.**

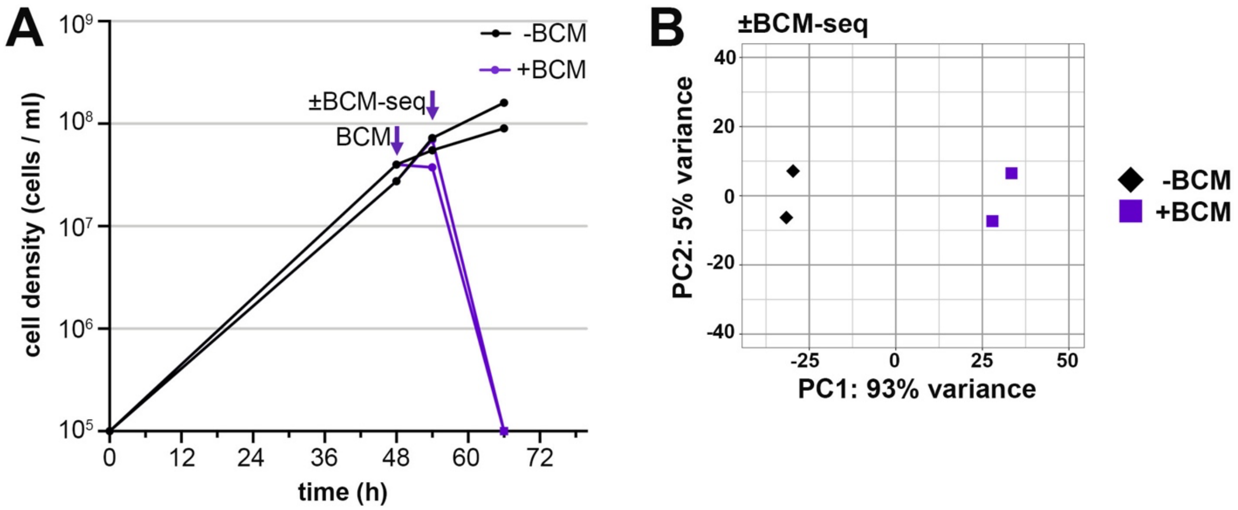

Fig. S5. Effect of BCM treatment on *B. burgdorferi* growth and analysis of BCM-seq data. **(A)** Growth curve of ±BCM cultures. Cells (PA003) were grown to a density of 4.0×10^7 cells/ml after dilution of the starter culture, the culture split and half treated with BCM for 6 h; n=2. Cell growth was monitored by dark field microscopy enumeration at the indicated time points. Each data point (circles) represents an individual biological replicate (the same cultures used for the ±BCM-seq analysis). The square data points represent a timepoint with no visible cells. **(B)** Principal component analysis plot to show correlation among total -BCM and +BCM RNA-seq replicates. For each sample, read counts were quantified at annotated genes and normalized by variance-stabilizing transformation. Normalized counts were used in the principal component analysis to estimate the relationships between samples.

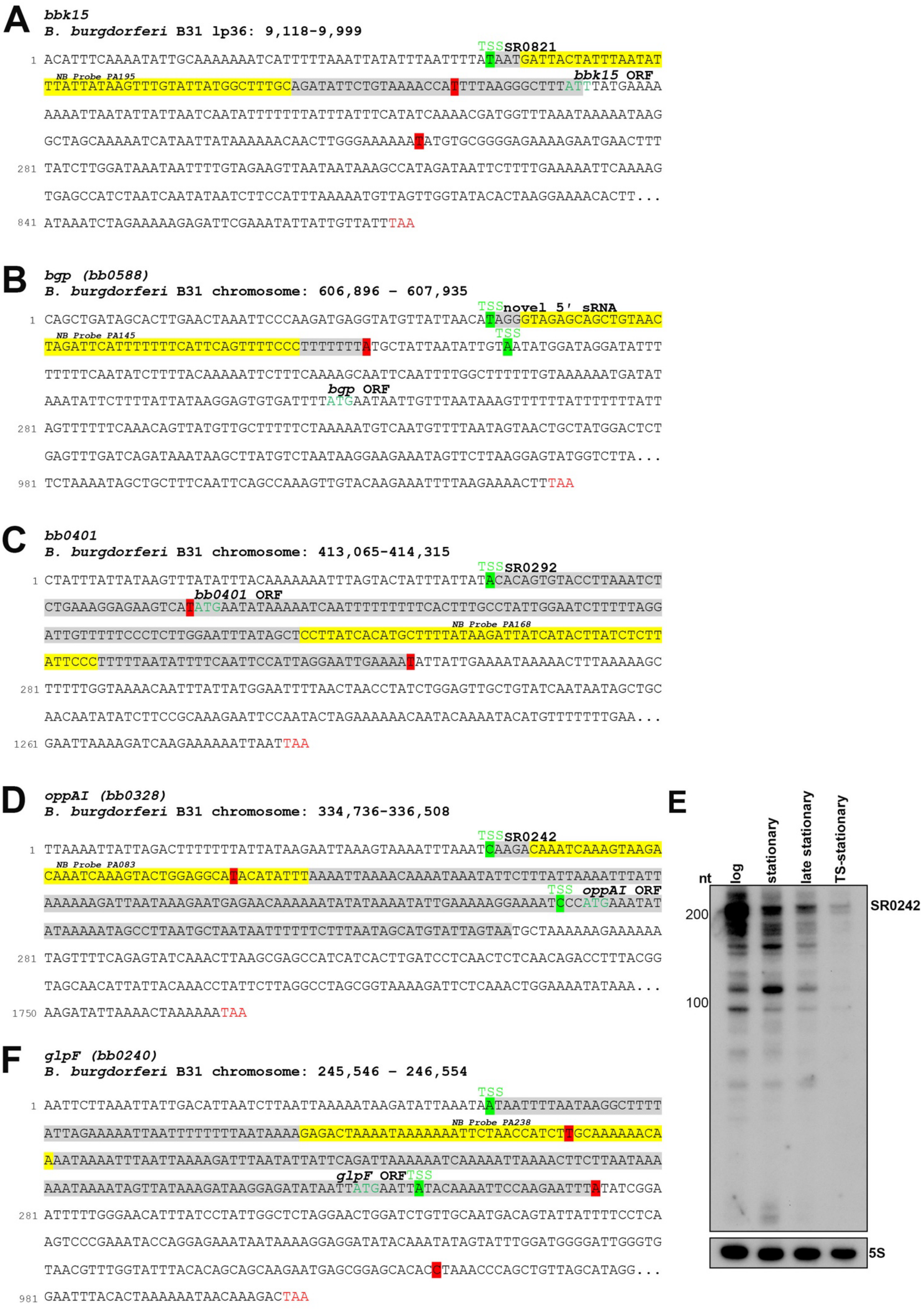

A
bbk15
B. burgdorferi B31 lp36: 9,118-9,999
TSS SR0821
1 ACATTTCAAAATATTGCAAAAAAATCATTTTTAAATTATATTTAATTTTATAATGATTACTATTTAATAT
NB Probe PA195
bbk15 ORF
TTATTATAAGTTTGTATTATGGCTTTGCAGATATTCTGTAAAACCATTTTAAGGGCTTTATTTATGAAAA
AAAATTAATATTATTAATCAATATTTTTTTATTTATTTCATATCAAAACGATGGTTTAAATAAAAATAAG
GCTAGCAAAAATCATAATTATAAAAAACAACTTGGGAAAAAATATGTGCGGGGAGAAAAGAATGAACTTT
281 TATCTTGGATAAATAATTTTGTAGAAGTTAATAATAAAGCCATAGATAATTCTTTTGAAAAATTCAAAAG
TGAGCCATCTAATCAATATAATCTTCCATTTAAAAATGTTAGTTGGTATACACTAAGGAAAACACTT...
841 ATAAATCTAGAAAAAGAGATTCGAAATATTATTGTTATTTAA
B
bgp (bb0588)
B. burgdorferi B31 chromosome: 606,896 – 607,935
TSS novel 5′ sRNA
1 CAGCTGATAGCACTTGAACTAAATTCCCAAGATGAGGTATGTTATTAACATAGGGTAGAGCAGCTGTAAC
NB Probe PA145
TSS
TAGATTCATTTTTTTCATTCAGTTTTCCCTTTTTTTATGCTATTAATATTGTAATATGGATAGGATATTT
TTTTTCAATATCTTTTACAAAAATTCTTTCAAAAGCAATTCAATTTTGGCTTTTTTGTAAAAAATGATAT
bgp ORF
AAATATTCTTTTATTATAAGGAGTGTGATTTTATGAATAATTGTTTAATAAAGTTTTTTATTTTTTTATT
281 AGTTTTTTCAAACAGTTATGTTGCTTTTTCTAAAAATGTCAATGTTTTAATAGTAACTGCTATGGACTCT
GAGTTTGATCAGATAAATAAGCTTATGTCTAATAAGGAAGAAATAGTTCTTAAGGAGTATGGTCTTA...
981 TCTAAAATAGCTGCTTTCAATTCAGCCAAAGTTGTACAAGAAATTTTAAGAAAACTTTAA
C
bb0401
B. burgdorferi B31 chromosome: 413,065-414,315
TSS SR0292
1 CTATTTATTATAAGTTTATATTTACAAAAAAATTTAGTACTATTTATTATACACAGTGTACCTTAAATCT
bb0401 ORF
CTGAAAGGAGAAGTCATATGAATATAAAAATCAATTTTTTTTTCACTTTGCCTATTGGAATCTTTTTAGG
NB Probe PA168
ATTGTTTTTCCCTCTTGGAATTTATAGCTCCTTATCACATGCTTTTATAAGATTATCATACTTATCTCTT
ATTCCCTTTTTAATATTTTCAATTCCATTAGGAATTGAAAATATTATTGAAAATAAAAACTTTAAAAAGC
281 TTTTTGGTAAAACAATTTATTATGGAATTTTAACTAACCTATCTGGAGTTGCTGTATCAATAATAGCTGC
AACAATATATCTTCCGCAAAGAATTCCAATACTAGAAAAAACAATACAAAATACATGTTTTTTTGAA...
1261 GAATTAAAAGATCAAGAAAAAATTAATTAA
D
oppAI (bb0328)
B. burgdorferi B31 chromosome: 334,736-336,508
TSS SR0242
1 TTAAAATTATTAGACTTTTTTTATTATAAGAATTAAAGTAAAATTTAAATCAAGACAAATCAAAGTAAGA
NB Probe PA083
CAAATCAAAGTACTGGAGGCATACATATTTAAAATTAAAACAAAATAAATATTCTTTATTAAAATTTATT
TSS oppAI ORF
AAAAAAGATTAATAAAGAATGAGAACAAAAAATATATAAAATATTGAAAAAGGAAAATCCCATGAAATAT
ATAAAAAATAGCCTTAATGCTAATAATTTTTTCTTTAATAGCATGTATTAGTAATGCTAAAAAAGAAAAAA
281 TAGTTTTCAGAGTATCAAACTTAAGCGAGCCATCATCACTTGATCCTCAACTCTCAACAGACCTTTACGG
TAGCAACATTATTACAAACCTATTCTTAGGCCTAGCGGTAAAAGATTCTCAAACTGGAAAATATAAA...
1750 AAGATATTAAAACTAAAAAATAA
E
nt
log
stationary
late stationary
TS-stationary
200
100
SR0242
5S
F
glpF (bb0240)
B. burgdorferi B31 chromosome: 245,546 – 246,554
TSS
1 AATTCTTAAATTATTGACATTAATCTTAATTAAAAATAAGATATTAAATAATAATTTTAATAAGGCTTTT
NB Probe PA238
ATTAGAAAAATTAATTTTTTTTAATAAAAGAGACTAAAATAAAAAAATTCTAACCATCTTGCAAAAAACA
AAATAAAATTTAATTAAAAGATTTAATATTATTCAGATTAAAAAATCAAAAATTAAAACTTCTTAATAAA
glpF ORF TSS
AAATAAAATAGTTATAAAGATAAGGAGATATAATTATGAATTATACAAAATTCCAAGAATTTATATCGGA
281 ATTTTTGGGAACATTTATCCTATTGGCTCTAGGAACTGGATCTGTTGCAATGACAGTATTATTTTCCTCA
AGTCCCGAAATACCAGGAGAAATAATAAAAGGAGGATATACAAATATAGTATTTGGATGGGGATTGGGTG
TAACGTTTGGTATTTACACAGCAGCAAGAATGAGCGGAGCACACCTAAACCCAGCTGTTAGCATAGG...
981 GAATTTACACTAAAAAATAACAAAGACTAA

G ***potAB (bb0642, bb0641)***

***B. burgdorferi* B31 chromosome: 681,839 – 679,754**

```
  P_potA
1    CACTTGATATTCTACAAAATTTTGCAAAAGCAAGAAAATTAATCGGTAAAAAAAATGAACTTAACCTTGA
     AAAAGCATCAAAAATATTAATCAAAGAATTTAGAGAGGGTAAATTTGGCAAAATAATTCTTGATAAGAAT
                                                                 TSS
     TATAATGCCTTTTAAAAAAGGCATTTACATAAATAATAATATTAAGTATAATCTTGATTTGTATTAATAC
                          potA ORF            NB Probe PA102
     AGCCATAAGGAGGTTGGAATTAGTTGGATAATTGTATCCTAGAGATTAAAAATCTAAGTCATTATTATGA
281  TAACAATGGAAACAAAACTTTAGATAACATAAATTTAAAAATTAAAAAAAATGAGTTTATCACACTACTA
     GGCCCATCCGGATGTGGAAAAACAACATTGATAAAAATATTGGGTGGTTTTTTAAGCCAAAAAAATGGAG
     AAATTTATTTCTTTTCTAAAGAAATATCTAAAACCAGTCCAAACAAAAGAGAAATTAATACTGTATTTCA
     AAATTATGCACTTTTCCCACATATGAATGTTTTTGACAATATTTCATTTGGACTTAGAATGAAAAAAACG
561  CCAAAAGATATAATCAAAGAAAAAGTAAAAACATCGCTTTCGCTGATAGGAATGCCAAAATACGCATACA
                                                 P_potB
     GAAATATTAACGAACTATCGGGGGGGCAAAAGCAAAGAGTTGCAATAGCAAGAGCAATGGTAATGGAACC
     TAAGCTTTTACTCCTAGATGAACCACTTTCCGCGCTTGATTTGAAAATGCGACAAGAGATGCAAAAAGAA
     TTAAAAAAAATACAGCGTCAGCTTGGAATCACATTCATATATGTTACTCACGATCAAGAAGAGGCATTGA
                                     TSS potB 5'1-561
841  CAATGAGTGACAGAATCGTTGTAATGAATGAAGGAATAATTCTGCAAATAGGAACACCTGAGGAAATTTA
     CAATGAGCCTAAAACAAAGTTTGTAGCCGATTTTATTGGAGAAAGCAATATTTTTGATGGAACATATAAA
     AAAGAGCTGGTTGTAAGTTTGCTTGGTCATGAATTTGAATGCCTTGACAAAGGATTTGAAGCTGAAGAAG
     CAGTTGACCTTGTAATACGCCCAGAAGATGTAAAACTACTTCCAAAAGGAAAAGGACATTTAAGCGGAAC
1121 TATAACATCAGCAATTTTTCAAGGAGTTCATTACGAAATGACTCTAGAAATCCAAAAAACAAATTGGATA
         5'proc potB 5'334-561
     GTTCAAAGCACAAGACTTACAAAAGTTGGAGAAGAAGTTGATATATTTTTAGAACCTGATGATATTCATG
                         potB ORF
     TTATGCATAAGGAATAATGGTTTTGAAAAAGTTGATATTAATCATATACTCCATATTCCTACTAACATTT
                 5'proc potB 5'485-561 (SR0541)           NB Probe PA086
     AGTATTCTTCCCTTACTAATAATAATTATTGCTTGGATTTTTAAATGAAAAAAACGAATTTACCATCTATA
1401 ATTTCATTGGACTTTTAAATCCAAGCTATCTTAATATTTTTTCAAGAAGTCTAAAACTCGCAACAATAGC
       NB Probe PA093
     AACAATTTTTTGCATTTTAATAGGCTATCCTGCCGCTTGGCTAATTTCATTATCAAAAAAAAGTGCTCAA
     AACAAATTAATAATCATGATAATACTTCCTATGTGGATAAATACATTACTTAGAACTTATGCCTGGATGA
     GAATACTTGGAAAAAAACGGATTCATCAACAACTTATTTGAAAAGATCGGAATTGGAACTTTAGATCTTCT
1680 TTATAATGAACAGGCTGTTACAATAGGCATGATATACAATTTTTTGCCTTTTATGATCTTGCCAATATAC
     ACGGGGCTTTTAAAAATTAAGCCAGAATATATTGAAGCATCACAAGATCTTGGAGCAAGAATGTGGCAAA
     TATTACTTTATATAAAAATACCACTAACACTCTCTTACCTGGCAACAGGAATAATTATGGTATTTATTCC
     TTCAATTACGGTATTTATCATTTCAGATTTGCTAGGAGGCTCTAAACAAATTTTAATAGGAAATCTAATA
1959 AGCAAACAGTTTCTCTTTATAGAAGACTGGAATACTGGGGCTGCAATTTCATTTATTTTAATGTTAGTAA
     TATTAATTTTTAATTTAATAATAATAAAATTAATGCGAAAAAATAATGGGGAGTAA
```

H

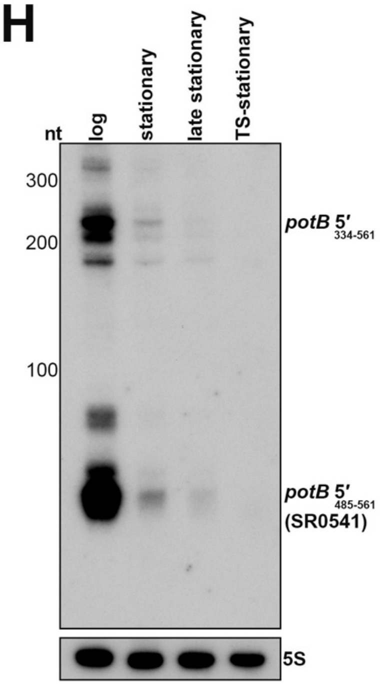

I

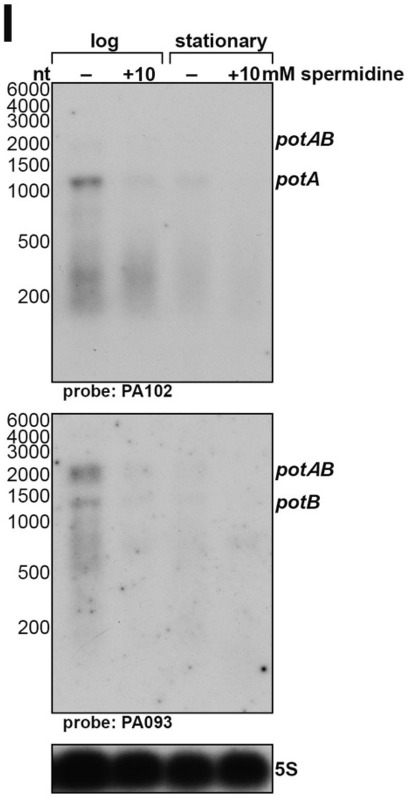

Fig. S6. 5′ mRNA sequences that harbor 3′ ends. DNA sequences for the **(A)** *bbk15* locus, **(B)** *bgp* locus, **(C)** *bb0401* locus, **(D)** *oppAI* locus, **(F)** *glpF* locus and **(G)** *potAB* locus are shown. Chromosome coordinates are indicated above the sequence for each locus. 5′ ends identified by 5′RNA-seq[1] are highlighted in green, TSSs or 5′ processed ends are indicated with text (TSS or 5′proc, respectively) over the highlighted nucleotide. 3′ ends identified by 3′RNA-seq, logarithmic phase condition (Supplementary Data 1), are highlighted in red. Dominant 5′ mRNA fragments, based on RNA-seq identified 5′ and 3′ ends and band sizes from the northern analysis (Fig. 6), are highlighted in grey and estimated sizes referred to in the main text. Annotated start and stop codons are labeled with green and red text, respectively. Probe sequences used for the northern analysis in Fig. 6 are highlighted in yellow. For panel G, *potA/B* promoter sequences used for luciferase transcriptional fusions in Fig. 7 are underlined with a wavy line, and the *potB* sequence used for luciferase translational fusions (*potB*$_{1\text{-}594}$) is underlined with a solid line. Northern analysis of SR0242 (**E**) and potB 5′ (**H**). Northern analysis was performed using the same RNA analyzed by agarose northern analysis in Fig. 6. Total RNA was separated on an acrylamide gel, transferred to a membrane and probed for the RNAs, sequentially on the same membrane. The 5S blot in panel E was repeated in panel H. **(I)** Northern analysis of effects of spermidine on *potAB* using a probe internal to the *potA* ORF (PA102; upper panel) or a probe internal to the *potB* ORF, downstream to the internal 3′ end (PA093; lower panel); performed on the same blot from Fig. 7B (RNAs probed sequentially on the same membrane). The 5S blot in panel I was repeated from Fig. 7B.

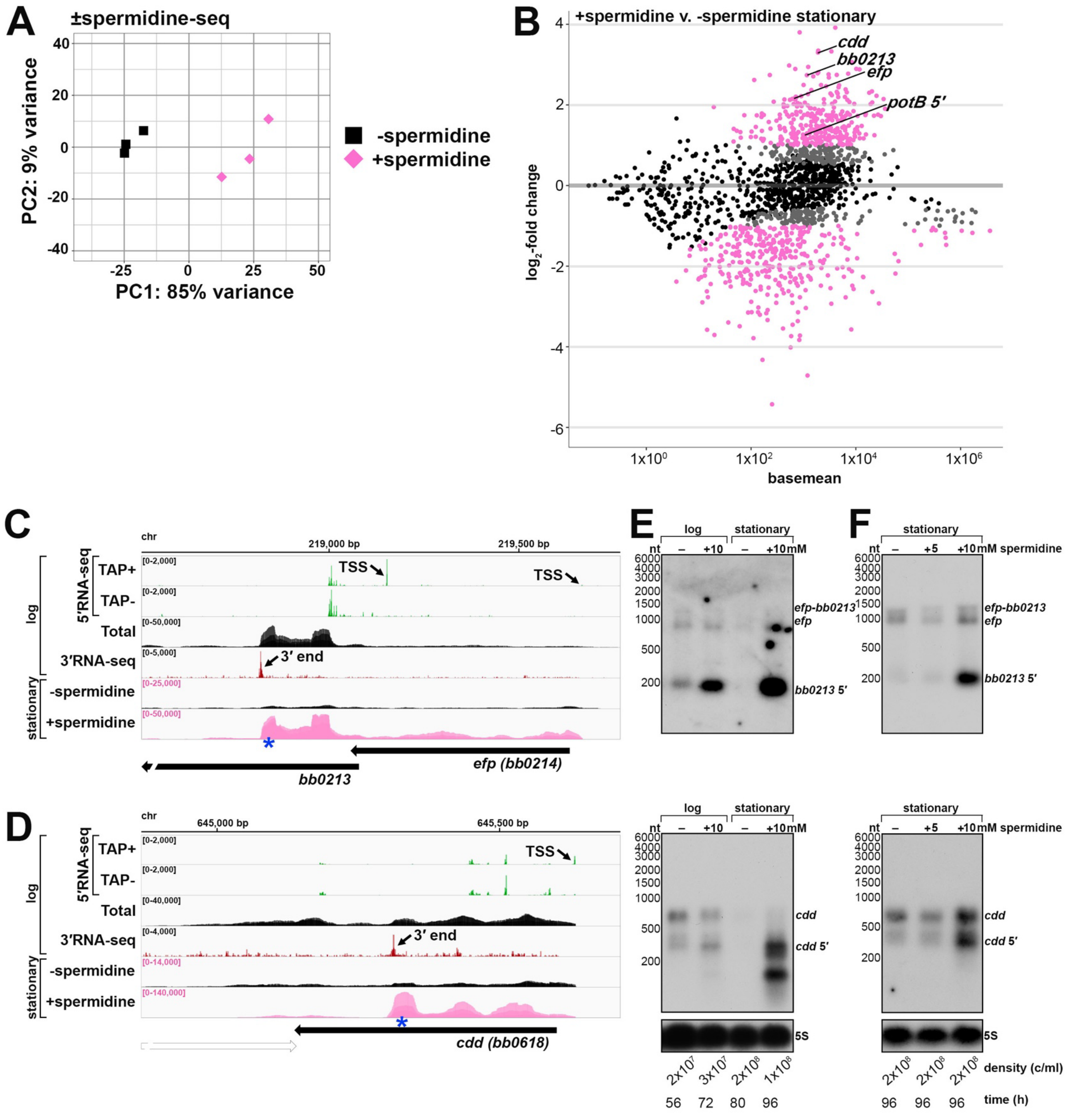

Fig. S7. Spermidine globally affects RNA levels. **(A)** Principal component analysis plot to show correlation among -spermidine and +spermidine total RNA-seq replicates. For each sample,

read counts were quantified at annotated genes and normalized by variance-stabilizing transformation. Normalized counts were used in the principal component analysis to estimate the relationships between samples. **(B)** MA-plot outputting results from DESeq2 analysis, comparing RNA levels in cells grown with/without the addition of 10mM spermidine in BSK-II. Three independent biological repeats of WT (PA003) were grown to stationary phase (as performed in Fig. 7B and described in the Methods) prior to cell lysis, RNA isolation and sequencing. Data are plotted by average $\log_2$-fold change (LFC) of annotated ORFs and sRNAs for +spermidine versus -spermidine (y-axis) over average normalized read count values ('basemean', x-axis). False discovery rate (FDR) was applied to two-sided Wald test p-values generated by DESeq2 to determine significance. Black points are non-significant ($p>0.01$), grey values are significant but have a LFC < 1.0, and pink points are significant with a LFC ≥ 1.0. RNA-seq browser image of the **(C)** *efp-bb0213* locus and **(D)** *cdd* locus. Browser image display total-, 5′- and 3′RNA-sequencing reads from logarithmic phase and ±spermidine stationary phase as in Fig. 7A. Scaling (pink text) for +spermidine is different from -spermidine to better visualize the -spermidine reads. **(E)** Northern analysis of effects of spermidine on *efp-bb0213* and *cdd* transcripts across growth, performed on the same blot from Fig. 7B (RNAs probed sequentially on the same membrane). The 5S blot in panel E was repeated from Fig. 7B. **(F)** Northern analysis of effects on *efp-bb0213* and *cdd* transcripts when exposed to 0, 5, and 10 mM spermidine performed on the same blot from Fig. 7C (RNAs probed sequentially on the same membrane). The 5S blot in panel F was repeated from Fig. 7C.

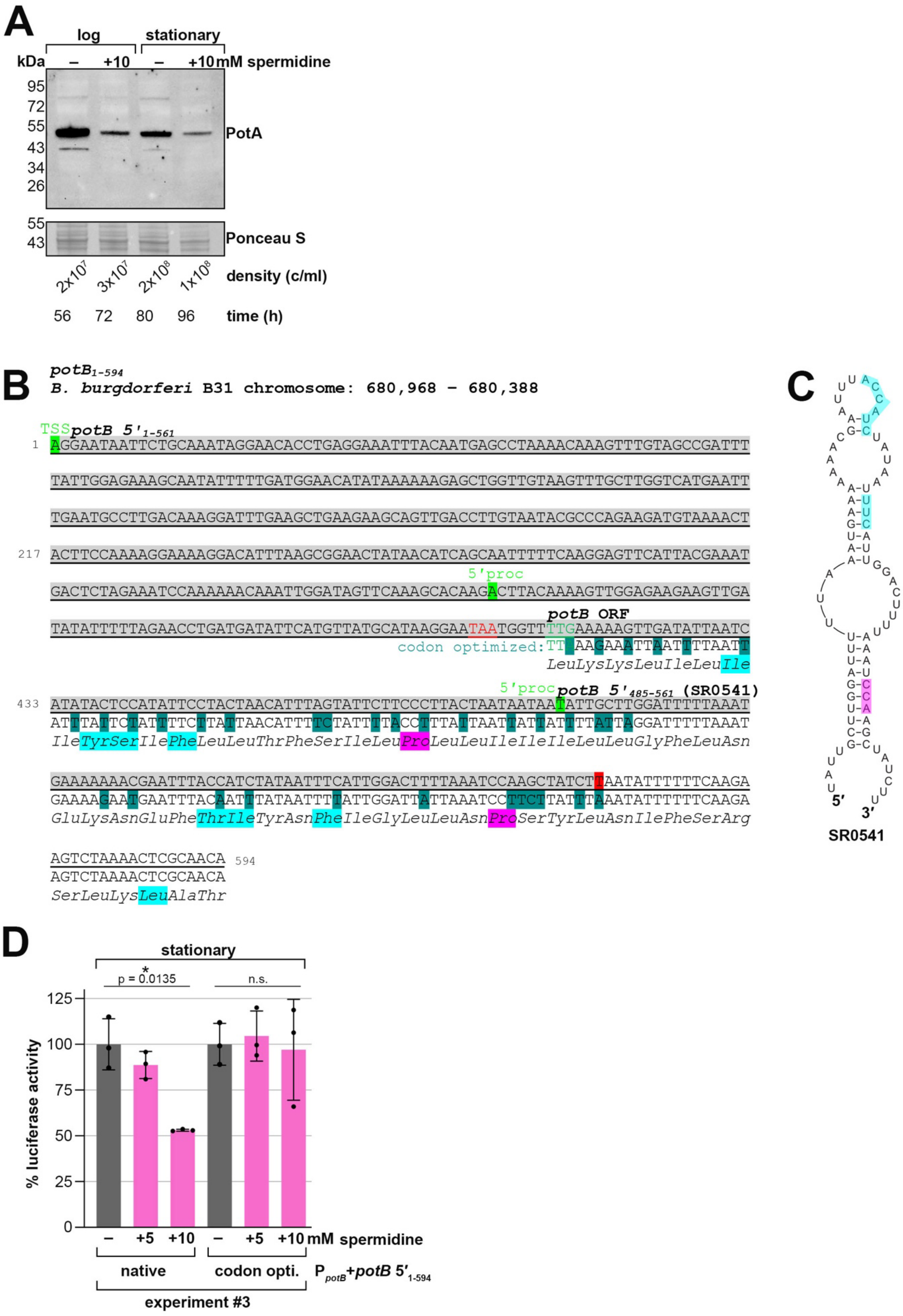
A
log
stationary
kDa
–
+10
–
+10 mM spermidine
95
72
55
43
34
26
PotA
55
43
Ponceau S
2x10^7
3x10^7
2x10^8
1x10^8
density (c/ml)
56
72
80
96
time (h)
B
potB 1-594
B. burgdorferi B31 chromosome: 680,968 – 680,388
TSS potB 5'1-561
1 AGGAATAATTCTGCAAATAGGAACACCTGAGGAAATTTACAATGAGCCTAAAACAAAGTTTGTAGCCGATTT
TATTGGAGAAAGCAATATTTTTGATGGAACATATAAAAAAGAGCTGGTTGTAAGTTTGCTTGGTCATGAATT
TGAATGCCTTGACAAAGGATTTGAAGCTGAAGAAGCAGTTGACCTTGTAATACGCCCAGAAGATGTAAAACT
217 ACTTCCAAAAGGAAAAGGACATTTAAGCGGAACTATAACATCAGCAATTTTTCAAGGAGTTCATTACGAAAT
5'proc
GACTCTAGAAATCCAAAAAACAAATTGGATAGTTCAAAGCACAAGACTTACAAAAGTTGGAGAAGAAGTTGA
potB ORF
TATATTTTTAGAACCTGATGATATTCATGTTATGCATAAGGAATAATGGTTTTGAAAAAGTTGATATTAATC
codon optimized: TTGAAGAAATTAATTTTAATT
LeuLysLysLeuIleLeuIle
5'proc potB 5'485-561 (SR0541)
433 ATATACTCCATATTCCTACTAACATTTAGTATTCTTCCCTTACTAATAATAATATTGCTTGGATTTTTAAAT
ATTTATTCTATTTTCTTATTAACATTTTCTATTTTACCTTATTAATTATTATTTTATTAGGATTTTTAAAT
IleTyrSerIlePheLeuLeuThrPheSerIleLeuProLeuLeuIleIleIleLeuLeuGlyPheLeuAsn
GAAAAAAACGAATTTACCATCTATAATTTCATTGGACTTTTAAATCCAAGCTATCTTAATATTTTTTCAAGA
GAAAAGAATGAATTTACAATTTATAATTTTATTGGATTATTAAATCCTTCTTATTTAAATATTTTTTCAAGA
GluLysAsnGluPheThrIleTyrAsnPheIleGlyLeuLeuAsnProSerTyrLeuAsnIlePheSerArg
AGTCTAAAACTCGCAACA 594
AGTCTAAAACTCGCAACA
SerLeuLysLeuAlaThr
C
5′
3′
SR0541
D
stationary
*
p = 0.0135
n.s.
% luciferase activity
125
100
75
50
25
0
–
+5
+10
–
+5
+10 mM spermidine
native
codon opti.
P potB +potB 5′1-594
experiment #3

Fig. S8. PotA levels decrease with spermidine exposure and features of the *potB*$_{1\text{-}561}$ sequence. **(A)** Western analysis of PotA levels. Cells were grown in the presence of 10 mM spermidine as in Fig. 7B. Samples were harvested at the indicated time points to best match cell density (indicated below each sample) and total protein isolated. Protein extracts were separated on a Tris-Glycine gel, stained using Ponceau S stain, and probed with α-PotA antibodies[7]. **(B)** DNA sequence of *potB*$_{1\text{-}594}$ (used for translational luciferase fusions), as in Fig. S6G. Amino acid sequence of PotB is included beneath the corresponding DNA sequence, with rare codons highlighted in blue and proline residues highlighted in magenta. The PotA stop codon is indicated with red text. The codon optimized sequence (used for the translational luciferase fusion in panel D) is displayed under the native sequence; nucleotide changes are highlighted in turquoise. **(C)** Structure of SR0541 (*potB 5′*$_{485\text{-}561}$) RNA as modeled by RNAfold Web Server[8]. Nucleotide sequences encoding rare codons and proline residues are highlighted in blue and magenta respectively, as in panel B. **(D)** Luciferase analysis of translational fusions harboring the *potB* 5′ sequence. *B. burgdorferi* containing the native translational (P_{potB}+potB $5'_{1\text{-}594}$; strain PA223) or codon optimized translational (P_{potB}+potB $5'_{1\text{-}594}$codon optimized; strain PA403) fusions to luciferase were grown and exposed to spermidine as described in Fig. 7C. Luminescence measurements were normalized to the cell density in each sample by OD_{600} (see Source Data file). Each data point (black circles) represents an independent biological replicate (n=3) as the percent luciferase activity relative to the average normalized luciferase units in the -spermidine condition for each construct. Bars represent the average luciferase activity across all biological replicates ± standard deviation. Average activity across samples were compared by one-way ANOVA with Šídák's multiple comparisons test, GraphPad Prism 9.4.1 (n.s., not significant), see Source Data file for full statistical analysis.

SUPPLEMENTARY REFERENCES

1 Adams, P. P. *et al. In vivo* expression technology and 5' end mapping of the *Borrelia burgdorferi* transcriptome identify novel RNAs expressed during mammalian infection. *Nucleic Acids Res* **45**, 775-792 (2017). https://doi.org:10.1093/nar/gkw1180

2 Shishkin, A. A. *et al.* Simultaneous generation of many RNA-seq libraries in a single reaction. *Nat Methods* **12**, 323-325 (2015). https://doi.org:10.1038/nmeth.3313

3 Dar, D. *et al.* Term-seq reveals abundant ribo-regulation of antibiotics resistance in bacteria. *Science* **352**, aad9822 (2016). https://doi.org:10.1126/science.aad9822

4 Adams, P. P. *et al.* Regulatory roles of *Escherichia coli* 5' UTR and ORF-internal RNAs detected by 3' end mapping. *Elife* **10** (2021). https://doi.org:10.7554/eLife.62438

5 Arnold, W. K. *et al.* RNA-Seq of *Borrelia burgdorferi* in Multiple Phases of Growth Reveals Insights into the Dynamics of Gene Expression, Transcriptome Architecture, and Noncoding RNAs. *PLoS One* **11**, e0164165 (2016). https://doi.org:10.1371/journal.pone.0164165

6 Kingsford, C. L., Ayanbule, K. & Salzberg, S. L. Rapid, accurate, computational discovery of Rho-independent transcription terminators illuminates their relationship to DNA uptake. *Genome Biology* **8**, R22 (2007). https://doi.org:10.1186/gb-2007-8-2-r22

7 Lin, Y. H. *et al.* Spermine and Spermidine Alter Gene Expression and Antigenic Profile of *Borrelia burgdorferi*. *Infect Immun* **85** (2017). https://doi.org:10.1128/IAI.00684-16

8 Gruber, A. R., Lorenz, R., Bernhart, S. H., Neubock, R. & Hofacker, I. L. The Vienna RNA websuite. *Nucleic Acids Res* **36**, W70-74 (2008). https://doi.org:10.1093/nar/gkn188
