# Supplementary material for: Extensive diversity in RNA termination and regulation revealed by transcriptome mapping for the Lyme pathogen Borrelia burgdorferi
Source: Nat Commun. 2023 Jul 4;14:3931. doi: 10.1038/s41467-023-39576-1 (PMC10319736; doi:10.1038/s41467-023-39576-1)
Supplement: Supplementary file 3 — Description of Additional Supplementary Files [file 41467_2023_39576_MOESM3_ESM.pdf]

Description of Additional Supplementary Files:

Supplementary Data 1. 3′ ends identified by 3′RNA-seq. Curated 3′ ends for B. burgdorferi B31 grown to logarithmic phase at 35°C or temperature-shifted (~23°C to 35°C) stationary phase. The data for each growth condition are displayed on a separate tab. The replicon, genomic coordinate of the 3′ end (‘3′ end position’), DNA strand, average RNA-seq read count of the 3′ end of biological triplicates, 3′ end classification (see Methods), the gene ID, NCBI locus tag, and gene name(s) for the classification (‘details’), the gene descriptions, the sequence surrounding the 3′ end (50 bp upstream and 10 bp downstream, 3′ end nucleotide red and bolded), and the distance of a primary 3′ end from the upstream mRNA (‘predicted 3′ UTR length’) make up the columns of the table. Each 3′ end was also given an intrinsic terminator score8, which includes an output of the Kinefold structure9 with possible Atract, hairpin, loop and U-tract. Intrinsic terminator scores ≥ 3.0 are suggestive of intrinsic termination. An ‘undefined’ intrinsic terminator score indicates one that could not be calculated because the sequence could not be folded into a recognizable secondary structure. An ‘impossible’ intrinsic terminator score indicates one that is too small to be accurately calculated.

Supplementary Data 2. Previously annotated B. burgdorferi sRNAs with defined 5′ and 3′ ends. The sRNA ID, based on the nomenclature from10, replicon, genomic coordinates as determined algorithmically in this study (‘annotated 5′ coordinate’ and ‘annotated 3′ coordinate’), and DNA strand are listed for each sRNA. Additional columns give the 5′ most coordinate, 5′ coordinate with the highest read count, possible TSS(s) and possible processed 5′ end(s) determined by visual inspection of 5′RNA-seq1 as well as possible 3′ end(s) determined by visual inspection of 3′RNA-seq (this study). The dataset where the sRNA was previously detected4,10 is indicated. See Methods for details of visual inspection.

Supplementary Data 3. Updated B. burgdorferi B31 gene annotations. The replicon and its NCBI reference number, DNA strand, genomic coordinate of the 5′ and 3′ ends, sequence length, classification, commonly used gene IDs (‘gene ID’), NCBI locus tag, and gene name(s) with gene descriptions from NCBI and UniProt make up the columns of the table. 5′ and 3′ UTR regions are predicted algorithmically using mRNA primary ends identified from 5′RNA-seq1 and 3′RNA-seq (this study), respectively. Characterized and/or identified genes not listed in NCBI or UniProt databases are also noted.

Supplementary Data 4. Identification of Rho termination regions using ±BCM-seq. The data for each replicate are displayed on a separate tab. All identified Rho termination regions are represented, defined as regions with at least one genomic coordinate with a significance score < 1e-300) to accurately report and indicates a highly significant score. The third tab in the table summarizes, for each gene, if a Rho region was found 800 nt downstream of or internal to all annotated ORFs/sRNAs for either (indicated by ‘rep1’ or ‘rep2’), both (indicated by ‘both’), or neither (indicated by a ‘0’) in replicate datasets.

Supplementary Data 5. Analysis of 3′ ends in 5′ UTRs and within coding sequences. 3′RNA-seq identified 3′ ends for B. burgdorferi B31 that were between 200 nt upstream of an ORF and the corresponding stop codon. The replicon, genomic coordinate of the 3′ end ('3′ end position'), DNA strand, average RNA-seq read count of the 3′ end of biological triplicates, 3′ end classification (see Methods), the gene ID for the classification ('details'), the location of the 3′ end relative to an ORF – upstream ORF or internal ('ORF classification'), the distance of the 3′ end from the gene's annotated start codon, and the gene annotation of the associated ORF ('upstream ORF/internal details') make up the columns of the table. Each 3′ end was also given an intrinsic terminator score (see Methods), Rho score (an assessment of whether it is in a Rho termination region), and spermidinedependent score (an assessment of whether its generation is impacted by spermidine treatment, see Methods). Intrinsic terminator scores ≥ 3.0 are suggestive of intrinsic termination. An 'undefined' intrinsic terminator score indicates one that could not be calculated because the sequence could not be folded into a recognizable secondary structure. Rho and spermidinedependent scores ≥ 2.0 are suggestive of Rho termination and spermidineaffected, respectively. An 'undefined' Rho score indicates one that could not be calculated due to an absence of reads in the ±BCM adjacent regions. An 'undefined' spermidine-dependent score indicates one that could not be calculated due to an absence of reads in the ±spermidine adjacent regions. A significance score of 'n/a' indicates that the Rho or spermidine-dependent significance score was too low (< 1e-300) to accurately report and indicates a highly significant score. No significance scores were calculated for 'undefined' scores.

Supplementary Data 6. RNA-seq analysis of transcript levels after exposure to spermidine. Differential expression analysis was carried out using DESeq2 with the annotated ORFs and sRNAs listed in Supplementary Table 3. The replicon, DNA strand, the gene ID, NCBI/UniProt locus tag, and gene name(s) for the classification ('details') and gene descriptions, basemean, log2-fold change, lfcSE (standard error of the log2-fold change) and adjusted p-value ('padj') make up the columns of the table. DESeq2 analysis was performed in two ways, one which included reads overlapping 2 or more features (columns labeled with the suffix (allowOverlap)), one performed with standard quantification parameters that included only reads overlapping a single feature. A basemean value of 0 indicates the absence of reads in the feature in either condition. No log2-fold change, lfcSE, or adjusted p-value were calculated for features with a basemean of 0.

Supplementary Data 7. List of strains together and plasmids (tab 1) as well as oligonucleotides (tab 2) used in this study
