# Supplementary material for: Extensive diversity in RNA termination and regulation revealed by transcriptome mapping for the Lyme pathogen Borrelia burgdorferi
Source: Nat Commun. 2023 Jul 4;14:3931. doi: 10.1038/s41467-023-39576-1 (PMC10319736; doi:10.1038/s41467-023-39576-1)
Supplement: Supplementary file 11 — Reporting Summary [file 41467_2023_39576_MOESM11_ESM.pdf]

nature portfolio

Corresponding author(s): Philip P. Adams

Last updated by author(s): May 26, 2023

Reporting Summary

Nature Portfolio wishes to improve the reproducibility of the work that we publish. This form provides structure for consistency and transparency in reporting. For further information on Nature Portfolio policies, see our Editorial Policies and the Editorial Policy Checklist.

Statistics

For all statistical analyses, confirm that the following items are present in the figure legend, table legend, main text, or Methods section.

n/a	Confirmed	
☐	☒	The exact sample size (*n*) for each experimental group/condition, given as a discrete number and unit of measurement
☐	☒	A statement on whether measurements were taken from distinct samples or whether the same sample was measured repeatedly
☐	☒	The statistical test(s) used AND whether they are one- or two-sided<br>*Only common tests should be described solely by name; describe more complex techniques in the Methods section.*
☒	☐	A description of all covariates tested
☐	☒	A description of any assumptions or corrections, such as tests of normality and adjustment for multiple comparisons
☐	☒	A full description of the statistical parameters including central tendency (e.g. means) or other basic estimates (e.g. regression coefficient) AND variation (e.g. standard deviation) or associated estimates of uncertainty (e.g. confidence intervals)
☐	☒	For null hypothesis testing, the test statistic (e.g. *F*, *t*, *r*) with confidence intervals, effect sizes, degrees of freedom and *P* value noted<br>*Give P values as exact values whenever suitable.*
☒	☐	For Bayesian analysis, information on the choice of priors and Markov chain Monte Carlo settings
☒	☐	For hierarchical and complex designs, identification of the appropriate level for tests and full reporting of outcomes
☒	☐	Estimates of effect sizes (e.g. Cohen's *d*, Pearson's *r*), indicating how they were calculated

Our web collection on statistics for biologists contains articles on many of the points above.

Software and code

Policy information about availability of computer code

Data collection

Public datasets were downloaded with sra-toolkit fastq-dump v2.9.1_1.

Data analysis

lcdb-wf v1.7 (lcdb.github.io/lcdb-wf/releases/tag/v1.7) was used to process raw total RNA or termseq RNA sequences, align the processed reads to the genome and quantify gene expression. lcdb-wf uses the following publicly available tools: cutadapt v2.3 to remove any adapters while performing light quality trimming; fastqc v0.11.8 and multiqc v1.10 for read quality assessment; fastq_screen v0.13.0 to look for common sequencing contaminants; BWA-MEM v0.7.17 to map reads to the reference genome; samtools v1.11 to remove multimapper reads; the subread package featureCounts v2.0.1 to quantify reads in genes; deepTools v3.2.1 to generate strand-specific mapping profiles. Differential expression was performed using raw counts provided to DESeq2 v1.30.0.

Termseq-peaks v0.2 https://github.com/NICHD-BSPC/termseq-peaks/releases/tag/0.2 was used to call the initial set of termination peaks. It relies on the scipy.signal v1.5.3 Python package. Subsequent peak filtering was performed with deepTools v3.2.1 and Bedtools v2.27.1, ran via pybedtools v0.8.0.

Peak postprocessing includes the following tools: Kinefold (http://kinefold.curie.fr/download/kinefold_long_static.tgz) was used to calculate putative intrinsic terminator scores, by simulating the kinetic folding of mRNA as transcribed by RNAP; heatmaps of GC content with the heatmap function from the seaborn package v0.11; terminator logos were generated using MEME v5.4; ucsc-tools v377 and trackhub v0.2.4 for building UCSC browser track sessions.

rhoterm-peaks (https://github.com/gbaniulyte/rhoterm-peaks) was used for the identification of Rho regions and spermidine-dependent 3' regions.

Luciferase activity values were compared by one-way ANOVA with Šídák's multiple comparisons test inGraphPad Prism 9.4.1.

For manuscripts utilizing custom algorithms or software that are central to the research but not yet described in published literature, software must be made available to editors and reviewers. We strongly encourage code deposition in a community repository (e.g. GitHub). See the Nature Portfolio guidelines for submitting code & software for further information.

Data

Policy information about availability of data

All manuscripts must include a data availability statement. This statement should provide the following information, where applicable:

- Accession codes, unique identifiers, or web links for publicly available datasets
- A description of any restrictions on data availability
- For clinical datasets or third party data, please ensure that the statement adheres to our policy

All data generated for this manuscript have been deposited under the accession number GSE222088. The SuperSeries is set to private; reviewer token can be requested. The SuperSeries is composed of the following SubSeries:
- GSE222084 Extensive diversity in RNA termination and regulation revealed by transcriptome mapping for the Lyme pathogen B. burgdorferi [bulk RNA-seq]
- GSE222085 Extensive diversity in RNA termination and regulation revealed by transcriptome mapping for the Lyme pathogen B. burgdorferi [BCM RNA-seq]
- GSE222086 Extensive diversity in RNA termination and regulation revealed by transcriptome mapping for the Lyme pathogen B. burgdorferi [SPD RNA-seq]
- GSE222087 Extensive diversity in RNA termination and regulation revealed by transcriptome mapping for the Lyme pathogen B. burgdorferi [3’RNA-seq]

SRA accession number of published datasets: E. coli PRJNA640168; P. aeruginosa ERR3258013- ERR3258015; and B. subtilis ERS1048762,
Data analysis: ERS1051962, ERS1051954,E RS1051963.

Source data for this paper have been submitted to Figshare (https://doi.org/10.6084/m9.figshare.22569205), which includes all uncropped and unprocessed scans with molecular weight markers labeled.

Human research participants

Policy information about studies involving human research participants and Sex and Gender in Research.

Reporting on sex and gender	not applicable
Population characteristics	not applicable
Recruitment	not applicable
Ethics oversight	not applicable

Note that full information on the approval of the study protocol must also be provided in the manuscript.

Field-specific reporting

Please select the one below that is the best fit for your research. If you are not sure, read the appropriate sections before making your selection.

☒ Life sciences ☐ Behavioural & social sciences ☐ Ecological, evolutionary & environmental sciences

For a reference copy of the document with all sections, see nature.com/documents/nr-reporting-summary-flat.pdf

Life sciences study design

All studies must disclose on these points even when the disclosure is negative.

Sample size	All experiments were performed in biological duplicate or triplicate. It has been shown that two or three replicates are sufficient for RNA-seq analysis using DESeq2 (PMID: 25246651; PMID:27022035).
Data exclusions	No data were excluded.
Replication	For all RNA-seq studies, northern analysis using independently derived samples were preformed to reproduce observations observed by sequencing. Number of repeats for each experiment are reported in the figure legends, when appropriate. We observed the same results across independent experiments (performed at least twice).
Randomization	No randomization of samples was used in our study. Observations from RNA-seq experiments drove which genes to further study.
Blinding	Investigators were not blinded. The investigators had no bias towards the outcome of the life study experiments and everything was

performed openly with eager anticipation for any results obtained from the life study experiments.

Reporting for specific materials, systems and methods

We require information from authors about some types of materials, experimental systems and methods used in many studies. Here, indicate whether each material, system or method listed is relevant to your study. If you are not sure if a list item applies to your research, read the appropriate section before selecting a response.

Materials & experimental systems

n/a	Involved in the study
☐	☒ Antibodies
☒	☐ Eukaryotic cell lines
☒	☐ Palaeontology and archaeology
☐	☒ Animals and other organisms
☒	☐ Clinical data
☒	☐ Dual use research of concern

Methods

n/a	Involved in the study
☒	☐ ChIP-seq
☒	☐ Flow cytometry
☒	☐ MRI-based neuroimaging

Antibodies

Antibodies used	α-PotA; peroxidase labeled α-mouse (GE Healthcare; Cat#NIF825)
Validation	PMID: 28052993 (α-PotA)

Animals and other research organisms

Policy information about studies involving animals; ARRIVE guidelines recommended for reporting animal research, and Sex and Gender in Research

Laboratory animals	Mice: C3H/HeN (Envigo). 6-8 week old mice were used for all experiments. Mice were housed at 72°F; 50-70% humidity with a 12/12 hour light/dark cycle.<br><br>Ticks: Ixodes scapularis (Oklahoma State University). Larval and Nymphal ticks were used for all experiments. Ticks were stored in a 23°C incubator under 98% humidity.
Wild animals	Study did not involve wild animals
Reporting on sex	Female mice were used for the B. burgdorferi infection.
Field-collected samples	Study did not involve field-collected samples
Ethics oversight	Protocols for all animal experiments were prepared according to the guidelines of the National Institutes of Health, reviewed and approved by the Eunice Kennedy Shriver National Institute of Child Health and Human Development Institutional Animal Care and Use Committee.

Note that full information on the approval of the study protocol must also be provided in the manuscript.
